# Metabolomic Signatures and Machine Learning Models for Distinguishing Alzheimer's Disease and Dementia with Lewy Bodies

**DOI:** 10.1002/alz70856_098539

**Published:** 2025-12-24

**Authors:** Dany Mukesha, Maite Sarter, Mélitine Dubray, Floris Durand, Seval Kul, Lucas Pham‐Van, Stéphanie Boutillier, Guillaume Sacco, Hüseyin Firat

**Affiliations:** ^1^ Firalis, Huningue, France; ^2^ Université Côte d’Azur, Cognition Behaviour Technology Lab (CoBTeK), Nice, France; ^3^ Amoneta Diagnostics SAS, Huningue, France; ^4^ Centre Hospitalier Universitaire de Nice, CMRR, Nice, France

## Abstract

**Background:**

Alzheimer's Disease (AD) and Dementia with Lewy Bodies (DLB) are difficult to distinguish clinically due to overlapping symptoms, resulting in delayed or incorrect diagnoses. This study uses metabolomic profiles from serum samples and machine learning models to identify key biomarkers that distinguish these conditions.

**Method:**

Serum samples from the ADDIA study (ClinicalTrials.gov NCT03030586), including 55 AD, 14 DLB, and 52 healthy control (HC) individuals, were analyzed using a targeted metabolomics approach. Metabolites were quantified with the AbsoluteIDQ® p400 HR kit (Biocrates Life Sciences AG) and analyzed via high‐resolution liquid chromatography‐mass spectrometry (LC‐HR‐MS). Machine learning models, including Lasso, Random Forest, and XGBoost, were trained for classification. APOE genotyping was performed using the CE‐IVDR APOEasy® kit and incorporated into the models.

**Result:**

AD patients exhibited a significantly higher prevalence of APOE e3/e4 and e4/e4 genotypes compared to DLB and HC (*p* <0.001). Lipid dysregulations, particularly in phosphatidylcholines, lysophosphatidylcholines, triglycerides, and sphingomyelins, were observed across all groups. Lasso identified 63 metabolites, which, combined with APOE, improved AUC for AD vs. DLB from 0.78 to 0.81. Similarly, the AUC for AD vs. HC increased from 0.78 to 0.83 with APOE, while no improvement was seen for DLB vs. HC (AUC=0.78).

**Conclusion:**

This study demonstrates the potential of metabolomic biomarkers in differentiating AD from DLB, with APOE improving classification accuracy for AD. Future efforts will focus on validating findings in larger cohorts and translating these findings into non‐invasive, cost‐effective tool set to reduce AD and DLB misdiagnosis.

